# Neurological Disturbances of Ciguatera Poisoning: Clinical Features and Pathophysiological Basis

**DOI:** 10.3390/cells9102291

**Published:** 2020-10-14

**Authors:** Killian L’Herondelle, Matthieu Talagas, Olivier Mignen, Laurent Misery, Raphaele Le Garrec

**Affiliations:** 1 University of Brest, School of Medicine, Laboratoire Interactions Epithéliums-Neurones (Univ Brest, LIEN), F-29200 Brest, France; killianlherondelle@gmail.com (K.L.); matthieu.talagas@chu-brest.fr (M.T.); laurent.misery@chu-brest.fr (L.M.); 2 Department of Dermatology, University Hospital of Brest, F-29200 Brest, France; 3 University of Brest, School of Medicine, INSERM U1227, Lymphocytes B et auto-immunité, F-29200 Brest, France; olivier.mignen@univ-brest.fr

**Keywords:** ciguatera, ciguatoxin, neurological, sensory, pathophysiology

## Abstract

Ciguatera fish poisoning (CFP), the most prevalent seafood poisoning worldwide, is caused by the consumption of tropical and subtropical fish contaminated with potent neurotoxins called ciguatoxins (CTXs). Ciguatera is a complex clinical syndrome in which peripheral neurological signs predominate in the acute phase of the intoxication but also persist or reoccur long afterward. Their recognition is of particular importance in establishing the diagnosis, which is clinically-based and can be a challenge for physicians unfamiliar with CFP. To date, no specific treatment exists. Physiopathologically, the primary targets of CTXs are well identified, as are the secondary events that may contribute to CFP symptomatology. This review describes the clinical features, focusing on the sensory disturbances, and then reports on the neuronal targets and effects of CTXs, as well as the neurophysiological and histological studies that have contributed to existing knowledge of CFP neuropathophysiology at the molecular, neurocellular and nerve levels.

## 1. Ciguatera: An Underreported and Misdiagnosed Disease Lacking Effective Prevention and Treatment

**Figure 1:**
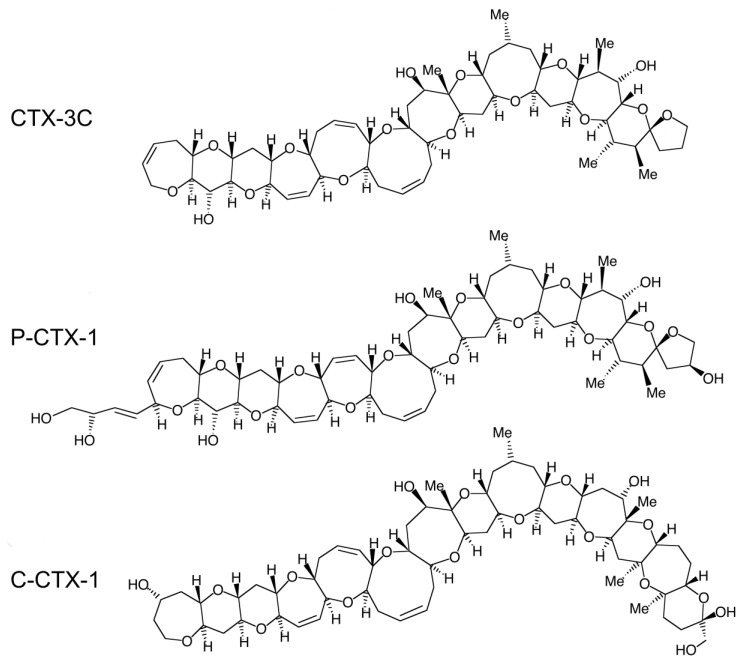
Structures of CTXs isolated from fish of the Pacific Ocean (CTX-3C and P-CTX-1) and the Caribbean Sea (C-CTX-1). The structure of the Indian-CTXs has not been characterized yet. From [7,11,31], respectively.

Ciguatera fish poisoning (CFP) is the most prevalent seafood poisoning worldwide, affecting mainly tropical and subtropical countries but also increasingly more temperate regions. Whereas this disease has existed for centuries, three major challenges remain: its prevention, diagnosis and treatment are poorly managed. CFP is caused by the consumption of reef fish belonging to usually edible species but which have been contaminated with ciguatoxins (CTXs). These toxins originate from benthic microalgae of the genus *Gambierdiscus* and *Fukuyoa* [1,2,3], which naturally populate coral reefs. Under the influence of poorly understood factors, these microalgae can proliferate and produce precursors of the congeners that intoxicate humans. Among these microalgal CTXs, which are called gambiertoxins (GTXs), CTX-4A [4,5], CTX-4B [5,6] and CTX-3C (Figure 1) [7,8] have been isolated from Pacific strains of *Gambierdiscus spp.* and herbivorous fish species involved in CFP outbreaks. Toxin-producing microalgae ingested by herbivorous grazing fish (of, e.g., the *Scaridae* or *Acanthuridae* families, which are especially incriminated in French Polynesia) are in turn eaten by carnivorous fish (such as species from the families *Muranidae*, *Serranidae*, *Lutjanidae* and *Carangidae*) [9]. During this transfer, GTXs are not only accumulated within the marine food chain but also biotransformed to become more polar and more toxic CTXs [10]. In the Pacific Ocean, the ultimate product of these toxifying biotransformations is Pacific-ciguatoxin-1 (P-CTX-1, also known as CTX1B), which is the most toxic for mammals of all CTXs known to date. Isolated from carnivorous fish from the Pacific Ocean [11], it is approximately 10-fold more potent in mice [10] and 50-fold more potent on the membrane potential of frog myelinated nerve fibers [12] than its microalgal precursor CTX-4B. In addition to fish, marine invertebrates such as giant clams have also been recently incriminated in ciguatera poisoning [13,14]. In addition to GTXs, which are lipid-soluble and therefore readily absorbed and slowly eliminated in fish and humans, *Gambierdiscus spp.* produce water-soluble toxins, including maitotoxins, gambierol and gambieric acids. However, until now there has been no evidence for their involvement in human CFP cases [15].

The distribution of ciguateric areas is sporadic (temporally variable), sometimes highly localized, and so far, unpredictable. Prevention of CFP cases could be achieved by detecting ciguateric fishes prior to consumption. This remains a challenge because CTX levels in fish that can cause adverse health effects in consumers are as low as parts per billion [16,17]. Ciguateric fish are indistinguishable from uncontaminated ones with regard to taste, smell, and appearance. CTXs are thermo- and relatively acid- and basic-stable toxins that are unaffected by cooking, salting or congealing [16]. Currently, there are no portable assays available to detect CTXs in fish in the field prior to consumption. To achieve this goal, the development of an enzyme-linked immunoassay (ELISA) is attractive for its accuracy, sensitivity, routine and portable use, and recent progress is promising [18,19,20,21]. Laboratory methods such as cell-based assays and HPLC-MS(/MS) are useful to detect, quantify and/or identify CTXs after suitable extraction from fish meal remnants [8,17,22,23,24,25,26,27] and mammalian fluids or tissues following intoxication [28,29,30]. Chemical methods are also used in a few laboratories to purify CTXs. Only small amounts of pure CTXs are available worldwide as chemical standards to monitor CTXs in marine foodstuffs or to confirm a CFP diagnosis and to study the pathophysiology of CFP disturbances.

CFP is a common disease in the Asia-Pacific region [32,33,34], Caribbean area [35] and Indian Ocean islands [36]. Several groups of CTXs have been isolated from fish from these regions, namely, Pacific (P-CTXs, including P-CTX-1, Figure 1) [6,11], Caribbean (C-CTXs, including C-CTX-1, Figure 1) [31,37,38,39] and Indian (I-CTXs) [40,41] CTXs, respectively. The global incidence of CFP is estimated to be 50,000–500,000 cases per year [16,36,42,43], with the highest incidences reported in Pacific Islands [44,45,46] (see [15] for details). The large range of incidence estimates takes into account the large number of underreported cases in endemic regions of CFP and the misdiagnosed cases in nonendemic areas [47]. The incidence of ciguatera in nonendemic areas is increasing with the increase in fish imports and tourism [48,49,50,51], and global warming has tended to extend the areas concerned [52,53,54,55]. CFP diagnosis in human cases is usually based on clinical signs, which are well known in endemic areas. The variety of CFP symptoms, and in some cases, their persistence (see Section 2) constitutes a challenge in its diagnosis for clinicians and suffering for patients. In nonendemic regions, misdiagnosis is frequent because CFP symptomatology is poorly recognized, especially the relapsing symptoms that can reoccur months after the ciguateric meal, thus making the link difficult to establish. CFP treatment is only symptomatic, with limited efficacy, especially for the most distressing sensory symptoms (see Section 4). This review first aims to describe the CFP clinical features, on which diagnosis is largely based, focusing on neurological symptoms, especially sensory disorders. The second part reports the neuronal targets and effects of CTXs, and the neurophysiological and histological studies that have contributed to the knowledge related to CFP neuropathophysiology at the molecular, neurocellular and nerve levels, which could be the basis for the development of specific treatments.

## 2. Clinical Features of Ciguatera

Clinically, CFP is a polymorphous syndrome. Furthermore, in some cases, the time course of its symptomatology is unusual. Indeed, in addition to disturbances that immediately follow the ingestion of the toxic fish, it is not rare that some symptoms reoccur months or even years after exposure.

### 2.1. Clinical Features of Acute CFP: A Variety of Neurological Signs with Pathognomonic Sensory Disturbances

The acute phase of CFP symptomatology includes a wide spectrum of symptoms, which have been well documented, especially in the Pacific, and to a lesser extent, in Caribbean regions. They comprise digestive, neurological, cardiac and general troubles that can occur in multiple combinations. Among this variety of symptoms, neurological and especially sensory disorders (abnormal sensations) predominate in all cases.

The first symptoms appear a few minutes to 24 h after the toxic fish meal, but occur between 1 and 6 h in 90% of cases [56]. Onset symptoms are typically circumoral and distal paresthesia (see details below) and/or a digestive syndrome, both of which develop early. Gastrointestinal disorders include nausea, vomiting, abdominal pain and diarrhea (watery stools). Neurological symptoms begin with early paresthesia on the face and especially around the mouth (lips, tongue), then rather involve the extremities. Pruritus (itch), which is a very common symptom, begins 1 to 2 days after the ciguateric meal, sometimes associated with a skin rash. Painful sensations on contact with cold (cold dysesthesia) appear mostly within the first 2 days. Myalgia, especially in the muscles of the legs, is also frequently reported, as well as other sensory disorders, including a metallic taste, feeling cold (which is associated with hypothermia), superficial hyperesthesia with sensations of burning and electric discharges, arthralgia mainly affecting the large joints (knees, ankles, shoulders and elbows), dental pain, headaches, painful micturition and other urogenital pain. Autonomic signs, including profuse sweating or hypersalivation, are also often described. Motor disorders, including muscle weakness and general asthenia, are common complaints [32,35,36,44,57,58,59,60,61].

Among the sensory disturbances, in most cases, three particular signs are characteristic: paresthesia, cold dysesthesia and pruritus. Associated with the knowledge of a fish meal followed by a digestive syndrome, these sensory disturbances allow for the diagnosis of ciguatera poisoning, i.e., are pathognomonic symptoms of CFP.

#### 2.1.1. Paresthesia

The first signs to appear, if there are no preceding digestive disorders, are circumoral paresthesia described as an unpleasant tingling, prickling and/or numbness around the lips as well as the nose, tongue and throat [62,63,64]. Intense paresthesia also affects the limbs, especially the extremities but also the arms and legs. Acral paresthesia does not develop as early as perioral paresthesia but belongs to the signs that may persist for one to several weeks after the incriminated meal [65,66,67].

#### 2.1.2. Cold Dysesthesia

With a high frequency regardless of the geographic area [68], and especially in the South Pacific (87% of 12,980 cases reported in French Polynesia) [32,60], this is one of the pathognomonic signs of CFP. Cold dysesthesia is described as painful tingling, burning, smarting, “electric shock” or “dry ice” sensations in the skin when it comes into contact with normally innocuous cold (cool water or objects), especially on the palms of the hands or soles of the feet [63]. The oral mucous membranes are also affected by cool drinks [44,69,70].

Although “paradoxical sensory disturbance” or “reversal of temperature perception” is frequently reported, gross temperature perception was found to be unaltered in a study devoted to this particular symptom [69]. Tingling and burning sensations to cold were triggered from 24 to 26 °C in five CFP patients whereas they normally appear below 15 °C and 4 °C, respectively [71]. Cold temperatures were perceived as burning or hot but different from the sensation evoked by noxious hot, and above all intensely painful. Of note, intradermal P-CTX-1 injection in human subjects has been shown to cause cold allodynia at concentrations higher than those inducing itch [72].

A functional magnetic resonance imaging (fMRI) study showed that brain areas responding to cold following intracutaneous injection of P-CTX-1 in human volunteers were located in the medial insula, the medial cingulate cortex, the secondary somatosensory cortex, frontal areas and the cerebellum [73].

#### 2.1.3. Pruritus

CFP is called “the itch” (la gratte) in the French-speaking South Pacific countries [32] but also “la gratelle” in the French West Indies [74], which reflects that itch is a common symptom. CFP pruritus is occasionally associated with a skin rash. It is first localized on the palms of the hands and the soles of the feet, which can be associated with peeling skin, and then it becomes generalized. Pruritus affects the limbs, the face or more diffuse areas, and is exacerbated at night [44,57,63,75,76]. Scratching lesions can become infected. CFP pruritus commonly persists or relapses for a long time, reoccurring especially after alcohol consumption or other factors that increase skin temperature or blood flow [77].

### 2.2. Cardiovascular and central Neurological Disorders in the Most Severe Cases

Cardiovascular disorders are not common and occur in only severe cases of CFP, in which the vital parameters need to be monitored. When present, cardiovascular disturbances occur within the first 24 h and usually consist of hypotension and bradycardia, or rarely tachycardia [78,79,80].

In severe CFP cases, in addition to peripheral neurological disturbances, symptoms reflecting abnormalities of the cranial nerves and cerebellar and central motor functions can occur. These include vertigo, visual and/or oculomotor disturbances, reduction or abolition of deep tendon reflexes, paresis and impaired motor coordination, with some cases developing an inability to stand or walk (ataxia). Ventilatory difficulties and distal cyanosis are present in the most severely affected individuals. Some cases of coma and death, although rare, have been described [32,49,57,81,82,83,84,85]. The CFP fatality rate has been estimated at 0.1–1%. Death is often associated with the consumption of ciguateric fish viscera, especially the liver, which contains higher levels of CTXs than the flesh [23,32,44,86].

### 2.3. Persistent and Relapsing Symptoms for Weeks, Months or Years

After acute exposure to CTX, digestive and cardiovascular disorders generally disappear within a few days, unlike the neurological and especially the sensory disorders. The persistence or relapse of these for weeks, months, and sometimes beyond, is undoubtedly one of the most distressing and disabling characteristics of CFP. These symptoms include pruritus, distal paresthesia, cold dysesthesia and arthralgia associated with fatigue [32,87,88]. The frequency of cases of so-called chronic ciguatera (i.e., chronic symptoms after acute exposure to CTX) has been estimated to be 5%–20% [88,89] but reached 34% in one study [90]. These symptoms persist intermittently without any particular triggering factor and/or reappear after the consumption of certain drinks or foods, especially alcohol but also peanuts, nonciguateric seafood, pork or poultry [64,66,91]. Occurring long after the incriminated meal, they make the diagnosis difficult, in particular for physicians unfamiliar with CFP.

In addition, unusual cases of chronic inflammatory conditions such as polymyositis and arthritis occurring months or years following CFP have been reported [92,93,94]. Whether these are related to CTX immune effects or immune parameter dysfunctions found in CFP patients remains to be clarified [95,96,97]. In addition, a few case reports or studies have documented repercussions on the mind and cognitive functions, including a tendency to develop depression, in CFP cases with chronic symptoms [98,99,100]. Such alterations have been observed in mice following repeated exposure to P-CTX-1 [101].

### 2.4. Factors Underlying the Variability in Ciguatera Symptomatology

Ciguatera symptomatology is polymorphous, and the nature and intensity of the symptoms described in case reports vary considerably. The factors behind this variability include the multiplicity of responsible toxins. The nature of the CTX congeners involved varies according to the fish trophic level (the transfer being associated with toxin bioactivation), age-related size (bioaccumulation) and species [61]. Some identified species are even popularly associated with a particular clinical sign, such as *Seriola fasciata* (lesser amberjack), which is known in the French West Indies to induce scalp pruritus and hair loss, and is thus called “the hairdresser” [35]. Slight structural differences between Caribbean, Indian and Pacific Ocean analogs of CTXs may explain subtly variable clinical signs from region to region. However, early digestive syndrome, acute and chronic peripheral neurologic disorders, and cardiovascular symptoms in severe cases are reported regardless of the geographic area [35,68].

Symptom variability among individuals sharing a fish meal may be related to the toxin amount ingested, which depends on the portion size and the fish part ingested. Previous and especially repeated exposure increases the illness severity, symptom intensity and individual sensitivity [32].

## 3. Pathophysiological Basis of Ciguatera Neurological Disturbances

The primary molecular neuronal targets of CTXs were identified a long time ago and continue to be refined. Electrophysiological studies have been performed with a number of excitable cells, and several major effects of CTX have been documented at the neurocellular level (see Table 1). In addition, neurophysiological and histological studies complement the knowledge related to CFP neuropathophysiology.

### 3.1. Neuronal Molecular Targets of CTXs Resulting in Membrane Hyperexcitability

#### 3.1.1. Voltage-Gated Sodium Channels (Na_v_) As the Main Primary Targets

After being mistakenly identified as anticholinesterases [155,156], CTXs have since been identified as sodium flow-increasing toxins [157]. CTXs are now well known to bind with high affinity and specificity to the “receptor-site 5” (localized to the S6 segment of domain I and S5 segment of domain IV) of the alpha subunit of Na_v_ channels [158,159,160], which are highly expressed in excitable cells. Electrophysiological studies have demonstrated that low nanomolar concentrations of CTXs modify the biophysical properties of both tetrodotoxin-sensitive (TTX-s) and tetrodotoxin-resistant (TTX-r) Na_v_ channels. Alterations of TTX-s Na_v_ properties comprise a shift of the activation threshold voltage to more negative values associated with an impairment of their inactivation process [103,104,109,110,111,114,161,162]. Although only a few studies have been performed in TTX-r Na_v_ expressing preparations like sensory neurons, they have shown that TTX-r Na_v_ channels are also targeted by CTXs. Modifications of the TTX-r Na_v_ biophysical properties by CTXs include an increase in the recovery rate from inactivation [162] and a shift of the activation voltage-dependence of Na_v_1.8 to more negative values [111]. Using a high-throughput membrane potential assay in HEK293 expressing human Na_v_ isoforms activated by P-CTX-1 plus veratridine, recent data showed that the rank order potency of P-CTX-1 on human Na_v_ subtypes was Na_v_1.8 > Na_v_1.3 > Na_v_1.1 > Na_v_1.2 > Na_v_1.5 > Na_v_1.7 > Na_v_1.6 > Na_v_1.4 [112]. The same authors provided electrophysiological evidence that CTXs shifted the activation voltage-dependence of all human Na_v_ isoforms to more hyperpolarizing potentials, with a greater impact on Na_v_1.8 and Na_v_1.9 than TTX-s Na_v_. The most altered inactivation parameters included a hyperpolarizing shift of the voltage-dependence of steady-state fast inactivation for Na_v_1.9 and Na_v_1.2, and an increase in the inactivation time constant for Na_v_1.8 [112].

**Figure 2:**
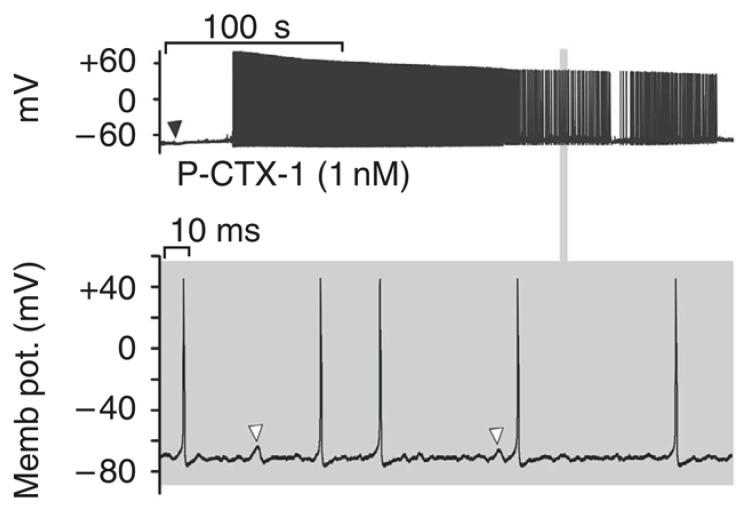
Spontaneous action potential firing induced by P-CTX-1 (1 nM) in cultured mouse dorsal root ganglion neurons. Upper panel: membrane depolarization followed by series of action potentials. Lower panel: expanded view showing membrane potential oscillations frequently followed by action potentials (reprinted from [111] with editor permission).

As a consequence, CTXs allow opening of the channel at the resting membrane potential and provoke a persistent Na^+^ conductance and a membrane depolarization, followed by spontaneous and repetitive firing in some models [103,104,107,108,111], including sensory neurons (Figure 2). It is worth noting that CTX binds both to resting and inactivated Na_v_ with similar affinity, and that during action potentials, the affinity of CTXs for Nav channels is increased, which has been proposed to be due to a channel conformation change [105]. CTX-induced depolarization of numerous excitable cells through activation of their Na_v_ channels was shown, including almost all types of peripheral nerves and muscles cell types (see Table 1), except smooth muscle cells on which only indirect depolarization or contraction through Na_v_ activation of innervation were shown in the tail artery, vas deferens and ileum [132,138,139]. It should be noted that CTXs were 10 to 100 times more potent in stimulating cardiac adrenergic nerves than myocardial cells in guinea pig atria [132].

Clinical acute and chronic features of CFP suggest that sensory afferents are the preferential targets of CTXs. Adult dorsal root ganglion (DRG) neurons express both TTX-s (mainly Na_v_1.6, Na_v_1.7 and Na_v_1.1) and TTX-r (Na_v_1.8 and Na_v_1.9) [163]. In mice, Na_v_1.6 and Na_v_1.7 are the main isoforms driving P-CTX-1-induced spontaneous dermal pain and action potential firing in cutaneous A-fibers, and cold allodynia is partly mediated by the sensitization to cold of TTX-sensitive A-fibers. Action potential firing in cutaneous and colonic C-fibers predominantly involves Na_v_1.8, which mediates cold allodynia and colonic visceral pain elicited by the toxin. Na_v_1.8 is also involved in the mechanical hypersensitivity induced in rats [111,112,164]. These findings are consistent with the roles of these isoforms in the firing properties of nociceptive neurons and cold pain [163,165,166].

The release of the neuropeptide calcitonin-gene related peptide (CGRP) induced by P-CTX-1 in mouse skin-nerve preparations was shown to involve Na_v_1.9 and the combined activation of Na_v_1.7 and Na_v_1.1 [144]. Although the Na_v_ isotypes mediating the pruritus induced by CTXs remain to be identified, the roles of Na_v_1.7 and Na_v_1.9 have been revealed in acute itch from other etiologies [167,168,169,170,171]. Interestingly, Na_v_1.7 is required for the release of another neuropeptide, substance P (SP), in the spinal cord from electrically stimulated DRG neurons, and the subsequent wind-up, i.e., increased dorsal horn neuron excitability following repeated C-fiber stimulation [172]. Na_v_1.9 activates at potentials close to the resting membrane potential and leads to a persistent sodium current that amplifies and prolongs the response to subthreshold inputs in DRG neurons, thus increasing their excitability [163,173]. Although Na_v_1.9 is mainly expressed in nonpeptidergic sensory neurons, it is also present in a subpopulation of peptidergic neurons [169], and the activation of a low channel density is sufficient to modify the neuronal electrogenic properties [173,174].

#### 3.1.2. Blockage of Voltage-Gated Potassium Channels (K_v_)

In addition to their activation of Na_v_ channels, CTXs, and especially GTXs, have been shown to inhibit voltage-gated potassium channels (K_v_), thus further increasing the membrane excitability. Among the CTXs isolated from ciguateric fish, P-CTX-1 partially blocked K^+^ currents in a dose-dependent manner in cultured rat myotubes [110]. P-CTX-1 also blocked K_v_ in rat DRG neurons, especially delayed-rectified and A-type potassium channels, without changing the voltage dependence of activation. This effect contributed to the prolonged action potential and afterhyperpolarization induced by the toxin [113].

Gambiertoxins and related toxins produced by *Gambierdiscus* microalgae (e.g., gambierol) exhibit actions on voltage-gated ion channels that differ from that of the congeners isolated from fish. CTX-4B, which was 50-fold less potent than its metabolite in fish (P-CTX-1) in inducing spontaneous action potentials in myelinated nerve fibers [12], was four-fold more potent than P-CTX-1 at inhibiting K_v_ channels, and two-fold more effective in affecting K_v_ than Na_v_ channels [175]. In mouse taste cells, high concentrations of gambierol failed to activate Na_v_ channels, whereas low nanomolar concentrations markedly inhibited K_v_ channels [176,177]. In contrast, in the same model, CTX-3C did affect Na_v_ channels, but it had no effect on K_v_ channels [178].

### 3.2. Neurocellular Effects of CTXs

A number of cellular effects induced by CTXs, including swelling, neurosecretion, an increase in intracellular calcium levels and the modulation of gene expression, have been demonstrated. All were found to be secondary to Na_v_ activation.

#### 3.2.1. Cell Swelling

Nanomolar concentrations of P-CTX-1, C-CTX-1, and CTX-3C induced approximately two-fold swelling of the nodes of Ranvier in frog myelinated nerve fibers. The molecular and cellular events proposed to explain this swelling are an osmotically driven water entry subsequent to the increased intracellular concentration of Na^+^ due to continuous Na^+^ influx through persistently activated Na_v_ channels. Indeed, nodal swelling of myelinated nerve fibers was prevented by tetrodotoxin and completely prevented and reversed by pretreatment with or the addition of hyperosmolar external solutions of D-mannitol [104,116,179]. More recently, this nodal swelling was found to involve both sodium influx and potassium efflux [117]. Interestingly, this swelling also occurred in sensory neurons and was also reversed by hyperosmolar D-mannitol extracellular solutions [118]. Motor nerve terminals and perisynaptic Schwann cell soma of frog neuromuscular junctions (NMJs) exposed to CTXs were also swollen [115,119,120].

These experimental findings are consistent with the swelling of both peripheral and central nervous structures observed in human CFP cases. In two severe cases, edema of myelin fibers, sometimes intra-axonal, or of the adaxonal Schwann cell cytoplasm, were revealed in biopsied sural nerves [59,121]. The skin biopsy of a mild CFP case with typical neurocutaneous disorders revealed swelling of intraepidermal nerve fibers 2 months after CTX exposure [70]. Finally, three cases of intramyelinic cytotoxic edema in the corpus callosum revealed by brain MRI were reported [122,123].

In mice experimentally intoxicated with a single dose of CTX, non-myelinated nerves of the enteric nervous system (the myenteric Auerbach plexus and submucosal Meissner plexus) were swollen, as were synapses in the myenteric plexus and vas deferens smooth muscle layers. A swelling of cardiac myocytes was also present, likely resulting from a direct effect (activation of the myocardial Na_v_ channels) instead of an indirect action through activation of Na_v_ in cardiac autonomic nerves. Surprisingly, swelling also affected erythrocytes in the cardiac tissue [124,125]. However, cell swelling induced by CTXs in nonexcitable cells has also been reported. P-CTX-1 and C-CTX-1 were able to induce frog erythrocyte swelling [126,127]. In mice, repeated CTX exposure for 2 weeks induced the swelling of cardiac myocytes but also endothelial cells lining the heart capillaries [128]. Whether nonexcitable cell swelling by CTXs is a direct effect or a result of a primary effect on neighboring excitable cells remains to be investigated.

#### 3.2.2. Neuromediator Release Linked to Autonomic Dysfunctions and Sensory Disturbances

In various models, CTXs have been shown to induce the release of neuromediators (Table 1), primarily neurotransmitters from the autonomic nervous system and, more recently, neuropeptides from sensory neurons. Their release appears to be of great importance in most of the CFP autonomic dysfunctions and sensory disorders.

- Acetylcholine (ACh) release:

A variety of symptoms occurring in both mice and humans, including sweating, salivation, eye watering and diarrhea [32,44,61,76,180,181], suggest a cholinergic syndrome. Although Na_v_ channels drive CTX effects, atropine effectively relieves the gastrointestinal and cardiovascular disturbances (i.e., hypotension and/or bradycardia) in humans, experimental animals, and tissues [57,125,129,130,131,132,133,182,183] (see details in Table 1), suggesting the involvement of ACh and the muscarinic receptors. CTX induced diarrhea in mice only at high doses but not very high doses, which cause rapid death. Diarrhea was associated with increased intestinal mucous secretion and epithelial damage, but the underlying mechanisms were not studied [184,185].

In addition, motor nerve terminals of frog NMJs and myenteric nerves in mice were depleted of synaptic vesicles after CTX exposure [119,125]. Direct evidence for ACh release induced by P-CTX-1 has been shown in frog neuromuscular preparations, in which the toxin triggered Na_v_-dependent repetitive endplate potentials (e.p.ps), i.e., repetitive action potentials of skeletal muscle fibers caused by repetitive neurotransmitter release from motor nerve terminals [106]. Caribbean C-CTX-1 Na_v_-dependently caused ACh release in the same model [120]. These action potentials, which led to spontaneous uncoordinated muscle contractions [106], could be related to the muscle weakness experienced during CFP.

In human volunteers, intracutaneous injection of low nanomolar concentrations of P-CTX-1 in the forearm elicited a striking axon reflex sweating [72]. This reflects the stimulation of efferent cholinergic sympathetic skin nerves [186], which is likely responsible for the profuse sweating described in some CFP cases.

- Release of noradrenaline (NAd) and other catecholamines:

With the exception of piloerection, which may result from stimulation of the sympathetic adrenergic cutaneous innervation, the involvement of the autonomous adrenergic system in the CFP syndrome is not obvious. However, a Na_v_-dependent CTX-induced NAd release from guinea pig atria has been associated with positive inotropic and chronotropic actions [135]. Further studies conducted in human or guinea pig atrial and papillary muscle preparations demonstrated that the extrasystoles and inotropic effects of CTXs primarily involved an indirect action through NAd released from the cardiac sympathetic innervation, whereas high concentrations directly activated myocardial cells [105,132,136,137,161]. The same mechanism, i.e., the Na_v_-dependent release of NAd from presynaptic sympathetic nerves, is involved in the contraction of smooth muscle cells of the vas deferens [130,131,138,187] and rat tail artery [139,140]. In rat brain synaptosomes and bovine chromaffin cells, CTXs caused a Na_v_-dependent release of dopamine [102] and catecholamines [141,142], respectively.

- Neuropeptide release and role of peptidergic neurons in sensory disturbances:

In vitro, 82% of the mouse DRG neurons that responded to P-CTX-1 were CGRP-positive, whereas 12% of the responding neurons bound isolectin B4, i.e., were nonpeptidergic neurons [111]. These findings indicate that sensory peptidergic neurons, i.e., neurons expressing neuropeptides including CGRP or substance P (SP), are major targets of CTXs. In vitro, P-CTX-1 elicits a release of CGRP from the murine skin at low nanomolar concentrations [72] with a higher release rate from mouse than rat skin [144]. The latter study demonstrated the involvement of Nav1.9, Nav1.7 and Nav1.1 but not Nav1.8 in CGRP release from mouse skin-nerve preparations. Both CGRP and SP have been demonstrated to be released by P-CTX-2 at 10 nM in a model of sensory neurons cocultured with keratinocytes [145]. Those neuropeptides initiate the so-called cutaneous neurogenic inflammation and contribute to pain and itch [188,189,190]. In humans, the P-CTX-1-induced neurogenic action on peptidergic sensory fibers was evidenced after intracutaneous injection of concentrations as low as 1 nM, that potently induced a long-lasting axon reflex flare [72], which is consistent with the skin rash reported in 20–30% of CFP cases [191] and the vasodilatory actions of CGRP and SP.

The SP release elicited by P-CTX-2 in cocultured DRG neurons and keratinocytes was recently found to involve the activation of protease-activated receptor-2 (PAR2) [146], a G protein-coupled receptor expressed in peptidergic sensory neurons that plays a role in itch and pain [192,193,194]. PAR2 activation was at least mediated by the cysteine protease cathepsin S. Interestingly, P-CTX-2 induced a marked Na_v_- and PAR2-dependent increase in intracellular calcium concentration in DRG neurons and also in keratinocytes. Human epidermal keratinocytes, which express Na_v_ and are increasingly identified as itch and pain transducers [195,196,197,198,199,200], are thus directly targeted by CTXs. In the same study, they also strikingly potentiated the neuronal SP release in the coculture. These findings suggest that keratinocytes could contribute to the neurocutaneous effects occurring during CFP.

- Modulation of central neurotransmitter release:

CTXs caused a Na_v_-dependent release of γ-aminobutyric acid (GABA), but not glutamate, in rat brain synaptosomes and mouse cortical neurons [102,143]. In rats, P-CTX-1 shifted the balance between excitatory and inhibitory neurotransmitter levels in the motor cortex toward inhibition. This effect was suggested to account for the reduced electroencephalography activity induced in this area [201] and, together with GABA release, to contribute to chronic fatigue, weakness, and depression associated with CFP through increased inhibitory neurotransmission [143,201].

#### 3.2.3. Increase in Intracellular Calcium Concentration in Excitable Cells by Multiple Mechanisms

Exocytosis of neurotransmitter or neuropeptide vesicles is known to be initiated by an increase in intracellular calcium concentration ([Ca^2+^]_i_). Depending on the cellular models, CTXs have been shown to increase [Ca^2+^]_i_ by several mechanisms (reported in Table 1).

- Calcium influx through the Na^+^/Ca^2+^ exchanger (NCX):

NCX normally functions to extrude Ca^2+^ from cells, contributing to cell calcium homeostasis. In *Torpedo* cholinergic synaptosomes, P-CTX-1 elicited ACh release, which was dependent on external Na^+^ and prevented by Na_v_ or NCX blockade [134,147]. This suggests that, as a consequence of the CTX-induced Na^+^ entry, the exchanger reversed its functioning mode to extrude the excessive Na^+^, leading to [Ca^2+^]_i_ increase, which in turn triggered ACh release.

- Calcium mobilization from intracellular stores:

In contrast, no external Ca^2+^ was required for P-CTX-1 to induce Na_v_-dependent repetitive action potentials in frog skeletal muscle fibers through repetitive ACh release from motor nerve terminals [106]. This rules out a mechanism involving Ca^2+^ influx into nerve endings and suggests that Na_v_ activation elicited ACh release through Ca^2+^ mobilization from internal stores. Similarly, the Na_v_-dependent [Ca^2+^]_i_ increase elicited by P-CTX-1 in mouse neuroblastoma × rat glioma NG108-15 hybrid cells was mediated by mobilization from Ca^2+^ internal stores [119]. In these cells, P-CTX-1 prevented the [Ca^2+^]_i_ increase induced by bradykinin, suggesting that the Ca^2+^ stores mobilized were dependent on inositol triphosphate (IP3) [148]. A similar mechanism was involved in the P-CTX-1-induced depolarization in myotubes from rat skeletal muscle cells, since this was associated with a [Ca^2+^]_i_ increase independent of external Ca^2+^ and a Na_v_-dependent transient increase in intracellular IP3 [110]. In bovine chromaffin cells, a Na_v_-dependent mobilization from Ca^2+^ stores contributed to catecholamine secretion, but the stores involved were caffeine-sensitive [141], suggesting the involvement of ryanodine receptors in the P-CTX-1-induced mobilization.

**Figure 3:**
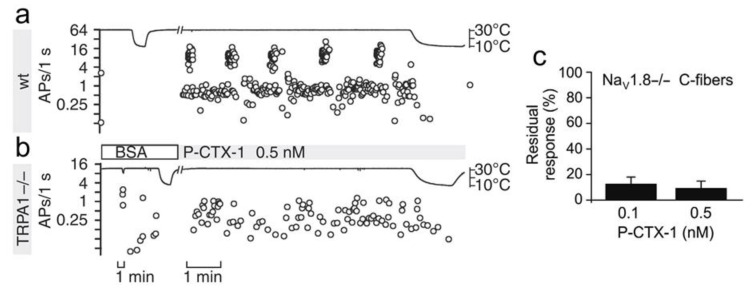
P-CTX-1 sensitizes C-type sensory fibers to cold in a Nav1.8- and TRPA1-dependent manner. Ongoing activity induced by P-CTX-1 (0.5 nM) in single C-fibers recorded from murine skin-saphenous nerve preparations from wild-type (wt) mice (**a**) and TRPA1-deficient mice (**b**). Residual cumulative action potentials induced in 5 min in single C-fibers from Nav1.8-deficient mice (**c**) compared with wt mice. Reprinted from [111] with editor permission.

- Calcium influx through transient receptor potential (TRP) channels:

TRP channels are calcium-permeable channels, among which the thermosensors TRP ankyrin 1 (TRPA1) and vanilloid 1 (TRPV1) are expressed in subpopulations of peptidergic sensory neurons and are involved in itch and pain [202]. In mouse DRG neurons, virtually all DRG neurons responding to P-CTX-1 (1 nM) by an increase in [Ca^2+^]_i_ were TRPA1-positive. The Na_v_1.8-dependent sensitization of TRPA1 to cold in C-fibers (Figure 3) contributed to P-CTX-1-induced cold allodynia in mice [111,164].

- Calcium influx through voltage-gated calcium channels (Ca_v_):

In addition, a [Ca^2+^]_i_ increase involving calcium influx through activated Ca_v_ could be a consequence of depolarization, if sufficient, induced by CTXs. Although P-type and N-type Ca_v_ have been ruled out in P-CTX-1 elicited ACh release from *Torpedo* cholinergic synaptosomes [134,147], L-type (Ca_v_1) and, to a lesser extent, N-type (Ca_v_2.2) channels, were shown to contribute to P-CTX-1-induced calcium responses in SH-SY5Y neuroblastoma cells [72]. Another study showed the ineffectiveness of L- and T-type Ca_v_ antagonists in preventing the P-CTX-1-induced CGRP release from mouse skin flaps, despite the reduction of the residual CGRP release after Na_v_ blockage [144].

#### 3.2.4. Modulation of Gene Expression

As a second intracellular messenger, [Ca^2+^]_i_ increase is able to initiate intracellular signaling pathways and lead to gene transcription. In yeast cells, CTX-3C and toxic *Gambierdiscus* extracts elicited a significant [Ca^2+^]_i_ increase and subsequent activation of the calcineurin signaling pathway [203], which contributes to transcriptional regulation in yeast and other eukaryotic cells. In RAW 264.7 macrophages, P-CTX-1 at low nanomolar concentrations induced the upregulation of inducible nitric oxide synthase (iNOS), tumor necrosis factor-α (TNF-α), interleukin (IL) IL-1β, IL-6 and IL-10 [149,150]. In mouse primary cortical neurons, CTX-3C exposure induced a Na_v_- and time-dependent expression modulation of a number of genes, including upregulation of the immediate early genes Arc and Egr and downregulation of the glutamate NMDA and AMPA receptors [114,151]. In mice, exposure to a high dose (median lethal dose) of P-CTX-1 downregulated immediate early genes and modulated the expression of genes involved in immune responses and detoxification in the blood, liver and brain [152,153,154]. In the brain, both downregulation of immediate early genes and anti-inflammatory gene signature have been proposed as neuroprotective mechanisms correlating with the hypothermia induced by P-CTX-1 [153]. Based on a whole blood transcriptomic profiling study, the activation of inflammatory pathways, as well as genetic variants of the adaptive immune system, were suggested to be involved in the persistence of CFP disturbances [96].

### 3.3. Neurophysiological and Nerve Histological Studies

Very few neurophysiological and histological studies have been performed in CFP cases. Most of them were severe forms of CFP, in which examinations revealed signs of demyelination. Rare studies have been carried out in experimentally intoxicated animals.

Allsop et al. [59] reported 3 CFP cases with central neurological disturbances in addition to marked sensory characteristic symptoms (severe cases). Neurophysiological studies performed on a peripheral mixed nerve (median nerve) revealed slowed motor conduction velocities in all cases. A more detailed study performed in one case also showed a slowing in sensory conduction velocity, a decreased sensory amplitude and delayed distal and F wave latencies, suggesting an altered myelin sheath. This was confirmed by a biopsy of the sural nerve, which revealed a striking edema in the adaxonal layer of the Schwann cell cytoplasm, with axonal compression and vesicular degeneration of the myelin.

A more recent study performed in a more typical CFP case reported no neurophysiological abnormalities in nerve conduction and needle electromyography studies. A skin biopsy from the distal leg, carried out 2 months after intoxication, revealed that the density of the intraepidermal nerve fibers was normal, but diffuse axonal swellings were observed [70].

In two cases diagnosed with acute polyradiculoneuritis after CFP, electromyogram recordings showed prolonged distal latencies, a decreased motor conduction velocity and absence of F wave. The muscle biopsy revealed a relative hypomyelination at the Ranvier node level [204].

A nerve conduction study performed in vivo in rat mixed and motor nerves of the ventral coccygeal nerve of the tail showed that CTXs induced a slowing of conduction velocities and delayed F wave responses, decreased action potential amplitudes, a prolonged absolute refractory period, and an increase in both the magnitude and duration of the supernormal period [205]. The same authors performed a neurophysiological study in 15 human cases of CFP presenting with acute gastrointestinal and sensory disturbances but also unusual motor disorders in the extremities. The sensory (sural) and motor (peroneal) conduction velocities were both decreased, but only significantly so in the sensory nerve. The action potential amplitude was increased only for the motor nerve. Absolute and relative refractory periods were markedly prolonged for both nerves [206].

A complete neurophysiological evaluation was performed in four cases of mild CFP with the usual clinical features. Motor and sensory conduction velocities in the peroneal and right median nerves, repetitive nerve stimulation of the right median nerve, as well as sudomotor sympathetic skin responses were normal. Only abnormalities of neuromuscular excitability assessed by a latent tetany test were noted [207].

An interesting axonal excitability study was performed in two patients presenting with relapsing sensory disturbances many years after the acute phase of CFP [91]. The results obtained by stimulating the median nerve revealed no abnormalities of axonal Na_v_ functions in both motor and sensory nerves. As noticed by the authors, given that the patients were asymptomatic at the time of the study, abnormalities during the period when the intermittent recurrent symptoms were occurring cannot be excluded.

As pointed out in the latter study, axonal excitability studies as well as conventional nerve conduction studies have investigated the functions of large-diameter myelinated but not small-diameter unmyelinated or thinly myelinated fibers, i.e., autonomic and sensory Aδ- and C-fibers, which are mainly targeted by CTXs. Abnormalities found in these neurophysiological studies may explain the muscular weakness or fatigability occurring during CFP but not the more common symptoms, including paresthesia, cold dysesthesia or pruritus involving the C- or Aδ-fibers.

### 3.4. Unsolved Issues: Prevalence and Persistence of Sensory Disturbances

Toxicokinetic data indicate that CTXs are readily absorbed and largely distributed to the body tissues, including the muscles, liver and brain (see [15] for details), likely due to their lipophilic nature. Although Na_v_ channels are highly expressed in all excitable cells, the predominance of sensory disorders suggests that CTXs particularly target somatosensory nerves/neurons. The time course of sensory disturbances (i.e., perioral paresthesia, abdominal pain and then pruritus and pain affecting the whole body including the face) [63] suggests an initial impact on trigeminal and enteric sensory afferents, then DRG and trigeminal sensory nerves/neurons. This is compatible with contact with/absorption through the mouth and intestine mucosa followed by distribution to the DRG and trigeminal fibers/neurons. In contrast to the neurons of the central nervous system, cell soma in the peripheral nervous system are located in ganglia and are not protected by blood–brain or blood–spinal cord barriers. Dorsal root and trigeminal ganglia are highly vascularized by fenestrated blood vessels, features that allow the preferential supply or even accumulation of a variety of neurotoxicants, favoring sensory neuropathy [208]. Likewise, peripheral nerves are partially protected by the blood-nerve barrier, which exhibits selective permeability. These toxicokinetic factors could contribute to the preferential sensory toxicity of CTXs. Consistent with this, peripheral nerves were the main tissues in which P-CTX-1 was quantified 2 h after intraperitoneal exposure, ahead of the intestine, kidneys, stomach, liver, heart and muscles [209].

Other toxicokinetic factors, including the quasi-irreversible binding of CTXs to Na_v_ and a potential release from binding sites (tissue or plasma proteins or lipoproteins), likely contribute to the persistence and reoccurrence of CFP sensory disorders. P-CTX-1 was indeed still detected in peripheral nerves 2 months after exposure [209]. However, CFP sensory disturbances can persist and/or reoccur many months or even years (see Section 2.3). The mechanisms involved remain to be clarified. In mice, the alterations induced in the anterior cingulate cortex one day after oral administration of a high dose (median lethal dose) of P-CTX-1 (neuronal spontaneous firings, an enhanced response to visceral noxious stimulation, synaptic potentiation, blockage of the induction of long-term potentiation, and reactive astrogliosis) were resolved by day 7 [210]. However, another study showed a recurrent motor strength deficit associated with neuronal apoptosis, reduced spontaneous firing rate and astrogliosis in the motor cortex 4 to 6 months after intraperitoneal administration of twice the same dose. The pinprick sensory test, which assesses functions of subpopulations of A-fibers and to a lesser extent C-fibers, showed no change in mice during 4 months following P-CTX-1 exposure compared with mice exposed to P-CTX-1 vehicle [211].

## 4. Treatment of Ciguatera Poisoning

So far, no clinically validated cure exists for CFP. Only symptomatic treatments are delivered to patients suffering from ciguateric signs, consisting of analgesics against muscle and joint pain and anti-histaminic drugs for the pruritus. Atropine appears to be effective in relieving the digestive and cardiovascular disorders [57,183].

In the Pacific, herbal remedies traditionally used to treat CFP [212,213] are still used currently [90]. Some of them were experimentally studied and showed:

- to speed up the recovery of mice from weight loss after intraperitoneal injection of ciguatoxic fish extract [214,215];
- to prevent and reverse the P-CTX-1-induced increase in nodal membrane excitability and swelling of frog myelinated axons [216];
- to inhibit the Na_v_ activation-based cytotoxicity [217] as well as to inhibit the LPS-induced nitric oxide production in RAW 264.7 macrophages [218,219], since P-CTX-1 was shown to induce such a production [127,149].

Some efficacy in the four aforementioned models was shown for *Heliotropium foertherianum* (formerly *Argusia argentea*, *Boraginaceae*), one of the most popular remedies in Polynesia and Melanesia but also in the Japan islands [214,216,217,218,220]. In addition, the plant extract and one of its active principles, rosmarinic acid, were able to displace a brevetoxin (a functional CTX analogue) from its Na_v_ binding site [221]. Rosmarinic acid also prevented the cytotoxicity induced by CTXs in human primary neurons from mixed brain cultures [222].

Some drugs were reported in a small handful of occasional cases to successfully relieve neurological symptoms, although they were never studied in randomized trials:

- Low dose of the tricyclic antidepressant amitriptyline during the first few days have shown a variable beneficial effect on some long-lasting neurological disturbances (paresthesia, myalgia, pruritus, headache) and bradycardia, and no effect on cold dysesthesia [223,224,225,226,227]. Experimentally, amitriptyline was ineffective in reducing cold allodynia induced in rats after intraplantar exposure to P-CTX-1 [72].
- Lidocaine is a local anesthetic that inhibits Na_v_ channels. In frog myelinated axons, lidocaine reversed the membrane hyperexcitability and axonal swelling induced by P-CTX-1 [12,216]. In vivo, it countered some cardiovascular effects and some peripheral nerve disturbances elicited by ciguatoxic extracts in cats [228] and in rats [229], respectively. In humans, beneficial effects on the persistent neurological signs were obtained in 3 patients by using the orally effective local anesthetic tocainide [225].
- Antiepileptic gabapentinoid drugs, including gabapentin [230] and pregabalin [231], were anecdotally but successfully used to treat persisting sensory disturbances in ciguatera patients.
- Nifedipine, a calcium channel antagonist, has successfully improved headache with no effect on myalgia, pruritus or cold dysesthesia [224]. It partially inhibited the P-CTX-1-induced calcium response in SH-SY5Y human neuroblastoma cells but failed to alter the cold allodynia induced by P-CTX-1 in rats [72].

The potential efficacy of other compounds has been reported in experimental studies. Brevenal, a polyether compound produced together with brevetoxins by *Karenia brevis*, is an antagonist of the binding of CTXs to Na_v_ [232,233]. It inhibited the P-CTX-1-induced catecholamine release from chromaffin cells [141,142]. Two monoclonal anti-CTX-3C antibodies were shown to synergistically neutralize in vitro and in vivo the toxic effects induced by this congener [234].

The most frequently used treatment in severe hospitalized cases is early intravenous perfusion of hypertonic D-mannitol (1 g/kg body weight as a 20% solution over 30–45 min), which is commonly used to reduce increased intracranial pressure associated with brain edema. Despite an efficacy demonstrated experimentally on nerve swelling and hyperexcitability [104,116,118,179,216], and in human cases [235,236,237], this treatment did not show an increased benefit relative to normal saline in a double-blind randomized trial [238] or any benefit when administered a few days after intoxication [239]. In addition, this treatment can worsen dehydration caused by the digestive disorders (diarrhea, vomiting) and patients should therefore be carefully monitored.

## 5. Conclusions

CFP is an underreported disease with a complex and polymorphous symptomatology, and the unique feature of a possible persistence or recurrence of disturbances long after the ciguateric meal. A precise knowledge of the “acute” and “chronic” clinical signs following acute exposure to CTX, among which sensory disturbances predominate, is necessary for CFP to be better recognized. This will allow for a better assessment of the global incidence of CFP as well as for providing appropriate medical support for intoxicated patients. Pathophysiological data indicate that the peripheral neurological and nonneurological symptoms arise from disorders of the sensory or autonomic nerves. The autonomic dysfunction-based disorders, including digestive and cardiovascular symptoms, resolve spontaneously or are treated effectively. Effective treatment to relieve the persistent sensory disturbances is lacking. The molecular mediators involved in their pathophysiology, such as Na_v_1.8 and TRPA1 in cold allodynia, could be the basis for developing specific therapeutics.

## Figures and Tables

**Table 1:** Electrophysiological and neurocellular effects of CTXs.

CTX Effect	Model	Reference
Na_v_-mediated depolarization ± spontaneous firing	Mouse differentiated neuroblastoma N1E 115 cells	[102]
	Frog myelinated nerve fibers	[103,104]
	Guinea pig atrial heart muscle cells	[105]
	Frog motor nerve terminals of NMJs	[106]
	Guinea pig sympathetic ganglia	[107]
	Rat parasympathetic neurons	[108,109]
	Rat skeletal myotubes	[110]
	Rat and mouse DRG neurons/afferents	[111,112,113]
	Mouse cortical neurons	[114]
Swelling	Ranvier nodes of frog myelinated nerve fibers	[104,115,116,117]
	Rat DRG neurons	[118]
	Frog motor nerve terminals	[115,119,120]
	Human adaxonal Schwann cell cytoplasm	[59]
	Human intra-epidermal and sural nerve fibers	[70,121]
	Human corpus callosum	[122,123]
	Mice myenteric plexus nerves	[124,125]
	Mouse and frog erythrocytes	[124,126,127]
	Endothelial lining cells of heart capillaries	[128]
Neuromediator release	ACh release from (parasympathetic innervation of):	
	Cat cardiovascular system	[129]
	Guinea pig and mouse small intestine, taenia caeci and ileum	[125,130,131,132]
	Frog atrial muscle	[133]
	Frog motor nerve terminals of skeletal NMJs	[106,120]
	Torpedo cholinergic synaptosomes	[134]
	NAd release from sympathetic innervation of:	
	Guinea pig and human atria	[105,132,135,136,137]
	Guinea pig vas deferens	[130,131,138]
	Smooth muscle of rat tail artery	[139,140]
	Catecholamines from bovine chromaffin cells	[141,142]
	Dopamine and GABA from rat brain synaptosomes	[102]
	GABA from mouse cortical neurons	[143]
	CGRP and/or SP from mouse and rat sensory neurons/afferents	[72,144,145,146]
[Ca^2+^]_i_ increase	Influx through NCX in Torpedo cholinergic synaptosomes	[134,147]
	Mobilization from internal stores:	
	Neuroblastoma x glioma hybrid NG108-15 cells	[106,148]
	Rat skeletal myotubes	[110]
	Bovine chromaffin cells	[141]
	Influx through TRPA1 in DRG neurons	[111]
	Influx through Ca_v_ in SH-SY5Y neuroblastoma cells	[72]
Modulation of gene expression	Upregulation of iNOS and pro-inflammatory cytokines in RAW 264.7 macrophages	[149,150]
	Gene expression modulation in mouse cortical neurons	[114,151]
	Expression modulation of genes involved in immune responses and detoxification in the blood, liver and brain	[152,153,154]
